# Epidemiology of revision hip replacement in Italy: a 15-year study

**DOI:** 10.1186/s12893-022-01785-8

**Published:** 2022-10-04

**Authors:** Umile Giuseppe Longo, Rocco Papalia, Giuseppe Salvatore, Salvatore Maria Tecce, Alexander Jedrzejczak, Martina Marcozzi, Ilaria Piergentili, Vincenzo Denaro

**Affiliations:** 1 Department of Orthopaedic and Trauma Surgery, Campus Bio-Medico University, Via Alvaro del Portillo, 200, Trigoria, 00128 Rome, Italy; 2 Research Unit of Orthopaedic and Trauma Surgery, Fondazione Policlinico Universitario Campus Bio-Medico, Via Alvaro del Portillo, 200, 00128 Roma, Italy; 3 Research Unit of Orthopaedic and Trauma Surgery, Department of Medicine and Surgery, Università Campus Bio-Medico di Roma, Via Alvaro del Portillo, 21, 00128 Roma, Italy

**Keywords:** Revision hip replacement, Italy, Epidemiology, Arthroplasty, Prevalence, Incidence

## Abstract

**Background:**

Over the past two decades, there has been an increase in the amount of primary total hip arthroscopies (THA) which in turn has increased the need for THA revision surgeries. The purpose of this study was to quantify the increase in THA revision in Italy, evaluate the causes and types of THA procedures performed.

**Methods:**

The data regarding revision hip prosthetic replacements performed both in public and private structures between 2001 and 2015 was collected by the National Hospital Discharge reports (SDO) carried out by the Italian Ministry of Health.

**Results:**

Overall, 109,746 Revision Hip Replacements (RHR) were performed in Italy from 2001 to 2015 in the adult population. The study shows a greater number of female patients underwent surgery between 2001 and 2015 and the 75- to 79-year age group had the highest incidence of THA revision. The main causes for THA revision were found to be “Mechanical complication of internal orthopedic device implant and graft” (31.5%), “Infection and inflammatory reaction due to internal joint prosthesis” (10.5%) and “Mechanical loosening of prosthetic joint” (8.3%).

**Conclusions:**

Revision hip replacement is growing and heavily affecting the population between 65 and 89 years and the main causes of THA revision have been quantified. The average length of hospitalization (LOS) was found to have decreased over the 14-year study period. Understanding the causes and risk factors for revision is essential in identifying avoidable complications and improving preventative care for patients undergoing primary implantation to decrease the revision burden.

## Background

Total hip arthroplasty (THA) is a highly successful surgical procedure with excellent reported long-term outcomes [1–4]. Over the past two decades, there has been an increase in primary hip replacement surgery across different countries [2, 5–7]. Although THA is a successful intervention for degenerative joint conditions, the increase in primary THA has led to an increase in prosthetic revision operations worldwide [8, 9]. Surgical indications for primary THA are extending to younger and more active patients which may contribute to the increased incidence of revisions as more patients outlive or wear out their implant [5, 6, 10–12]. Understanding the causes of THA and THA revision are not only important for improving patient outcomes, but information about national trends of hip replacements as well as public health costs are needed to establish healthcare and economic policies.

The most common indications for THA revision surgery have been reported to be aseptic loosening, infection, and dislocation [12–15]. In 2005, the Centers for Medicare & Medicaid Services and the National Center for Health Statistics implemented a series of changes to the International Classification of Diseases, Ninth Revision, Clinical Modification (ICD-9-CM) diagnosis and procedure codes related to failed total joint replacements and revision total joint arthroplasty procedures. These codes were updated to facilitate a better understanding of the mechanisms of failure following THA and to better quantify the types of THA revision procedures [10, 12].

The indication for revision surgery has a direct effect on cost, with infected cases being significantly more expensive than aseptic revisions [5]. Overall, revision surgery has a substantially higher average hospital cost and use of resources when compared to primary interventions [5, 16]. Revision surgeries take longer, use more expensive prosthesis, are associated with higher postoperative complication rates and longer hospital stays [5, 16].

The main purpose of this study is to quantify the number of patients who underwent THA revision surgery in Italy from 2001 to 2015 as well as comparing the diagnoses leading to the revisions. The secondary aim of this study is to evaluate the variation of patients stratified by age and sex in order to assess what kind of patient is most likely to undergo revision hip replacement surgery.

## Methods

An investigation of the National Hospital Discharge reports (SDO) reported to the Italian Ministry of Health for the years of 2001 to 2015 was conducted [17, 18]. The SDO collects anonymous data about the patient’s gender, age, domicile region, the region of hospitalization, length of the hospitalization stay, diagnoses and procedures. National population information was collected from the Italian National Institute for Statistics (ISTAT) for each year. Revision Hip Replacements (RHR) were defined by the following International Classification of Diseases, Ninth Revision, Clinical Modification (ICD-9-CM) major procedure code: 8153. An analysis of RHR in adult patients was performed. We defined “adult”—in agreement with ISTAT age classes—as patients aged at least 15 years.

### Statistics

All statistical analyses were performed using The Statistical Package for Social Sciences (SPSS) version 26 (Armonk, NY: IBM Corp.) and Excel (Microsoft) software. Descriptive statistical analyses (frequency and percentages for categorical data, mean and standard deviation for continuous data) were used. The incidence of the procedures was calculated as the number of surgeries divided by the size of the entire population of people ≥ 15-year-old in Italy (ISTAT data) and reported as the relative frequency per 100,000 residents.

## Results

### Demographics

**Fig. 1:**
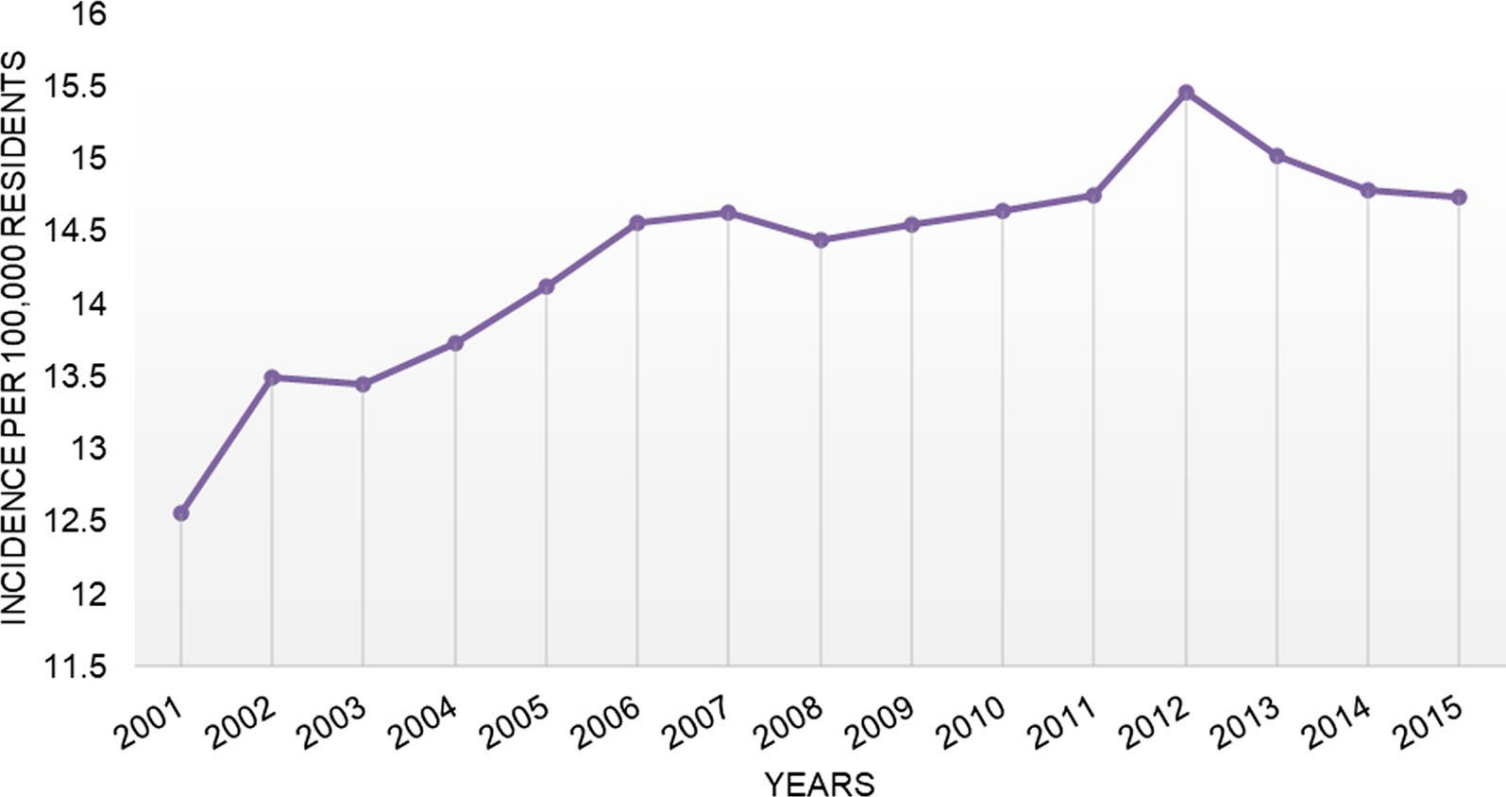
Incidence of THA revision per 100,000 residents from 2001 to 2015

**Fig. 2:**
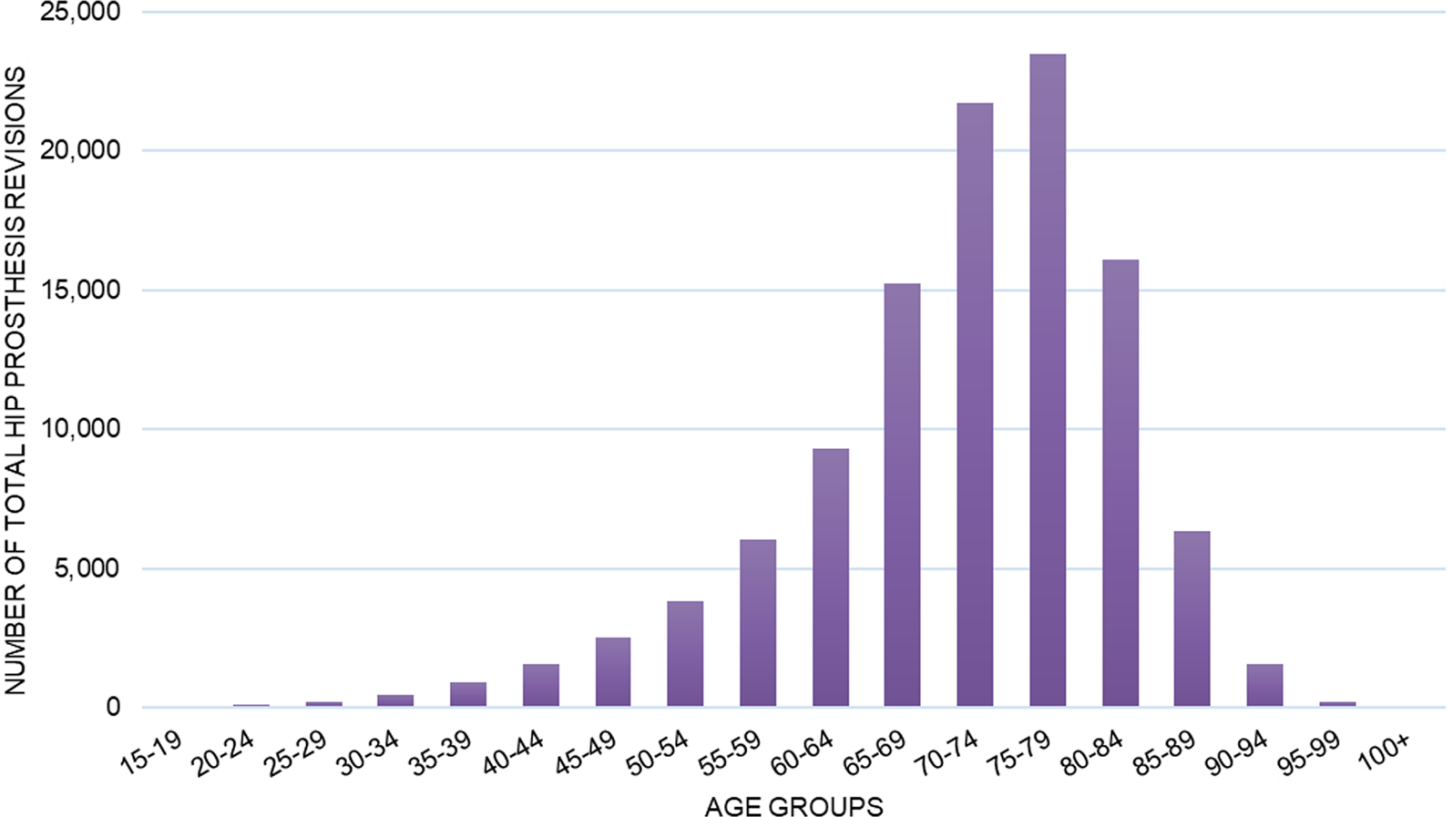
Number of THA revisions by age group

**Fig. 3:**
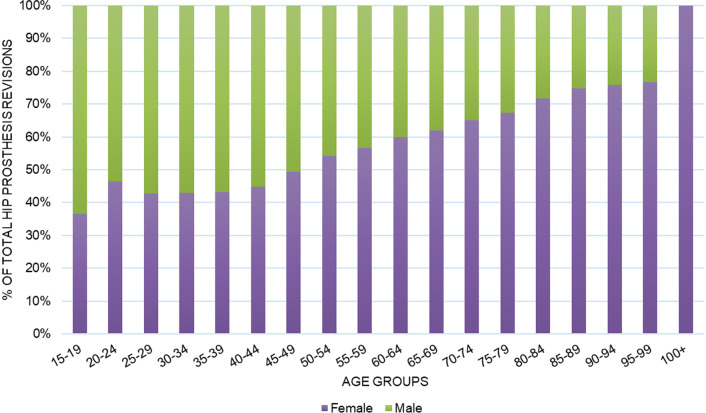
Gender breakdown of THA revision patients by age group

Overall, 109,746 RHR were performed in Italy from 2001 to 2015 on the adult population. The mean yearly incidence rate was 14.3 procedures for every 100,000 Italian inhabitants over 15 years of age. The incidence of operations increased from 12.6 in 2001 to 14.7 in 2015 per 100,000 person-years over 15 years old (Fig. 1). The 75- to 79-year age group shown a greater number of RHR (Fig. 2). The male/female ratio was 0.55, therefore, women represented the majority of patients undergoing RHR (females 64.6% and males 35.4%). Between the ages of 15 to 49 the patients were mostly male, while by the age of 50 the majority were female (Fig. 3). From 2001 to 2015, the average age of patients was 71 ± 11.3 years. During the entire period, the average age of females was always higher than that of males.

### Length of the hospitalization

**Fig. 4:**
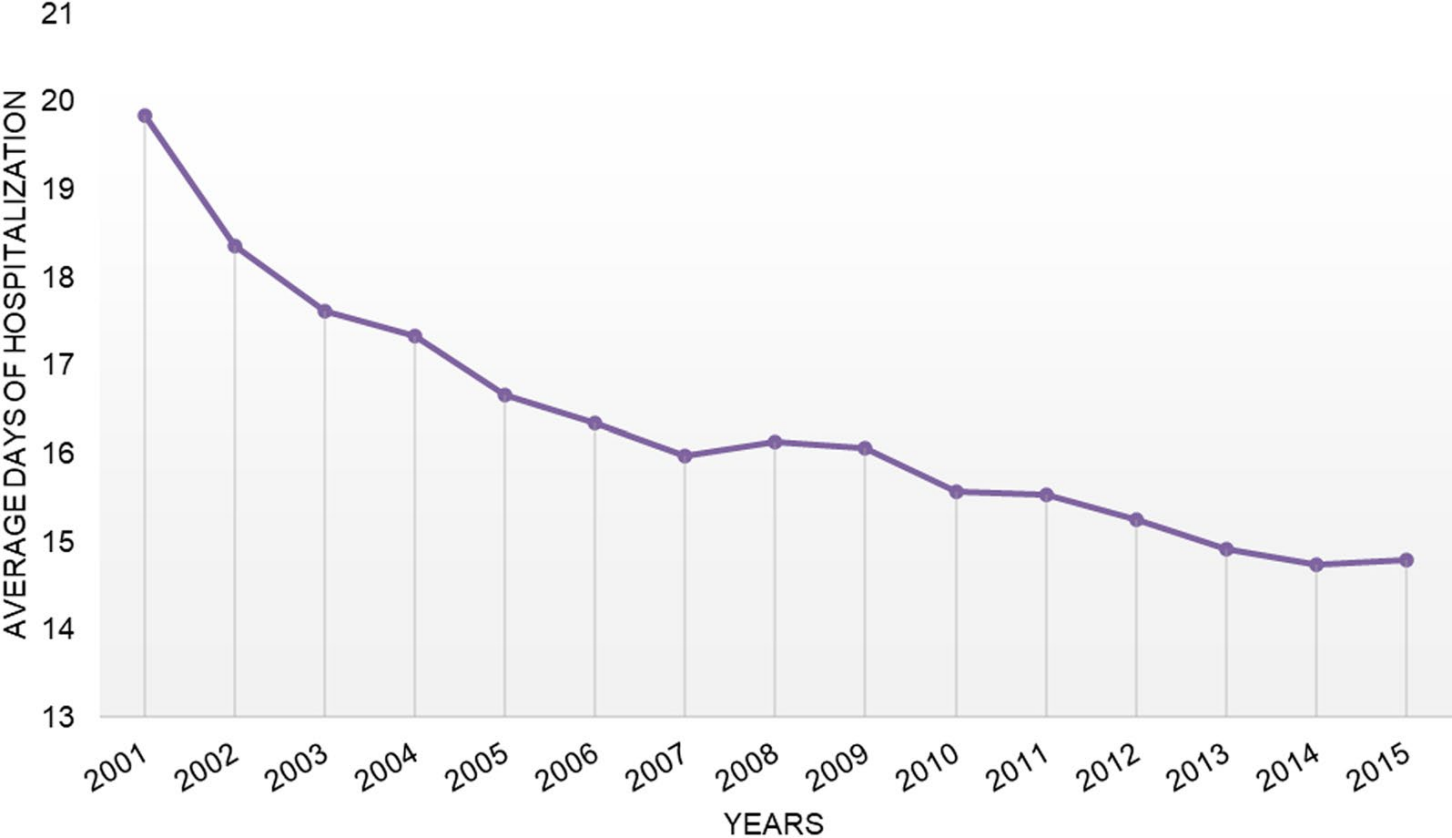
Average LOS

The median length of hospitalization (LOS) for these procedures was 16.24 ± 14.9 days, with a minimum of 0 and a maximum of 435 days. The trend of the average number of days of hospitalization was a decrease, from 19.83 ± 15.58 days in 2001 to 14.78 ± 15.48 in 2015 (Fig. 4). Females were hospitalized for more days than males (females 16.57 ± 15.05 days and males 15.65 ± 1.47 days). Older patients (≥ 60 years) had more days of hospitalization in respect to younger patients.

### Main primary diagnoses

**Fig. 5:**
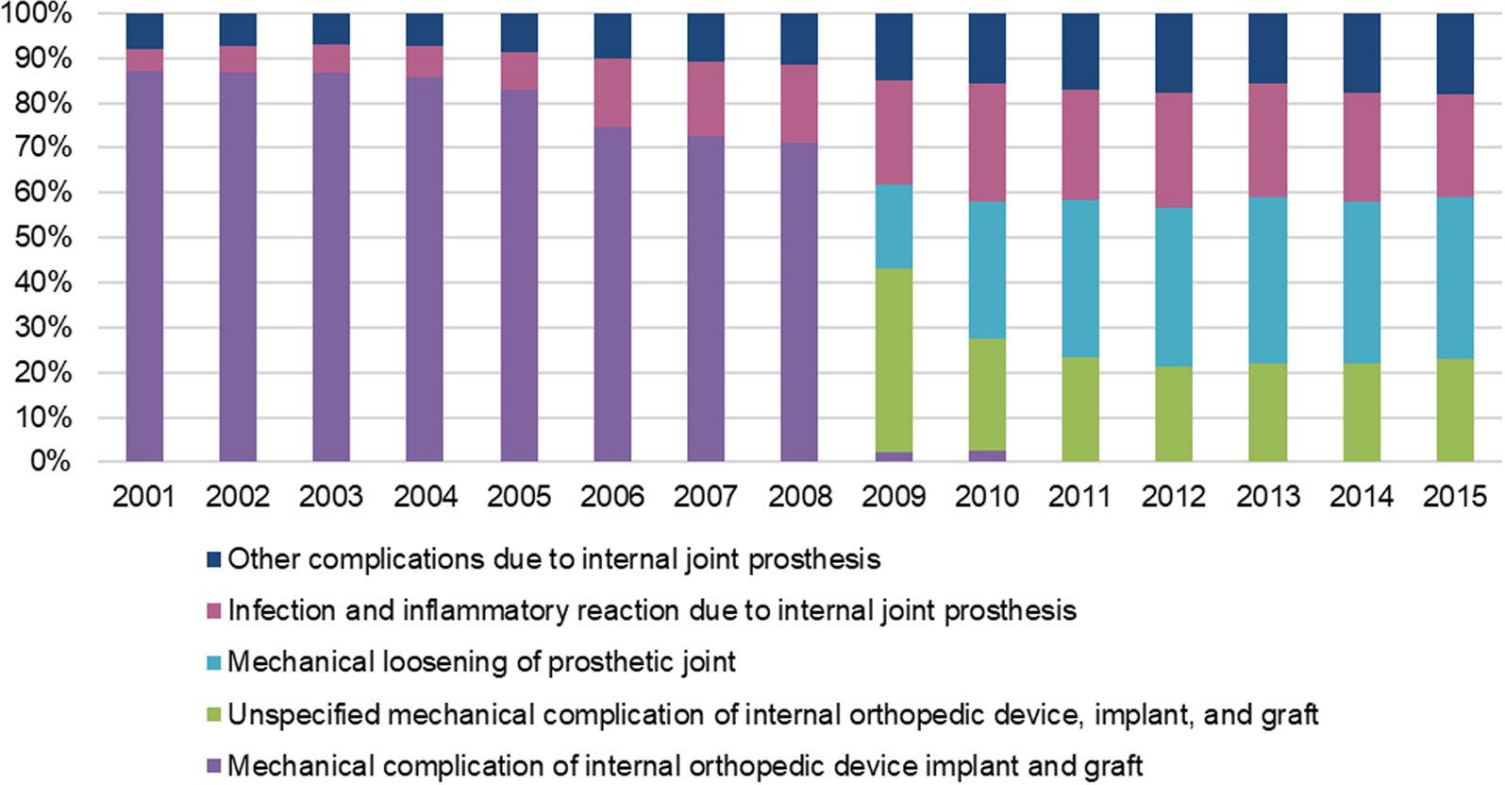
Most frequent causes for THA revision broken down by year

From 2001 to 2015, the main primary diagnoses were “Mechanical complication of internal orthopedic device implant and graft” (31.5%; diagnosis code: 9964), “Infection and inflammatory reaction due to internal joint prosthesis” (10.5%; diagnosis code: 996.66), “Mechanical loosening of prosthetic joint” (8.3%; diagnosis code: 996.41), “Other complications due to internal joint prosthesis” (7.8%; diagnosis code 99,677) and “Unspecified mechanical complication of internal orthopedic device, implant, and graft” (6.6%; diagnosis code: 99,640). From 2001 to 2008 the major primary diagnosis was “Mechanical complication of internal orthopedic device implant and graft”. In 2009 it was “Unspecified mechanical complication of internal orthopedic device, implant, and graft”, and from 2010 to 2015 it was “Mechanical loosening of prosthetic joint” (Fig. 5).

### Main primary procedures

Overall, the main primary procedures were “Revision of Hip Replacement, Not Otherwise Specified” (68.5%; procedure code: 81.53), “Revision of Hip Replacement, Both Acetabular and Femoral Components” (6.6%; procedure code: 70). “Revision of Hip Replacement, Acetabular Component” (7.6%; procedure code: 71), “Revision of Hip Replacement, Femoral Component” (4.5%; procedure code: 72), “Revision of Hip Replacement, Acetabular Liner and/or Femoral Head Only” (3.4%; procedure code: 73), “Arthrotomy for Removal of Prosthesis without Replacement, Hip” (2%; procedure code: 80.05) and “Total Hip Replacement” (2.5%; procedure code: 81.51).

## Discussion

Advances in bioengineering technology and prosthetic surgery have driven development of hip prostheses and increased the number of THA being performed [3]. The increase in performed THA has subsequently raised the frequency of THA revision surgery. Our data shows a net increase in THA revision procedures conducted in Italy between the years 2001 and 2015. We observed a consistent growth trend in the number of annual THA revisions performed, except for the years between 2008 and 2010 and after 2012 [6]. A decrease in incidence of THA revision surgeries was also observed from 2012 to 2015 [19]. The National Joint Registry in the UK reported similar growth in THA revisions until 2010, after which there was a decrease and plateau, however the overall trend from 2003 remains positive. Similarly the Swedish Hip Arthroscopy Register observed an increase in THA revisions until 2009 at which point the number plateaued until 2015 and then started to decrease [20]. In 2019 the number of revisions had dropped to 2123 reported THA revisions from its peak of around 2400 during the plateau [20].The reasons behind the observed decreases in THA revisions in the UK and Sweden are unknown, and could either be real or due to underreporting. The Australian Orthopedic Association (AOA) also found that the number of revisions has continually increased since 2002 but did not observe decrease until 2020, however, this was due to COVID-19 and is not expected to continue or affect the overall observed trend [21]. The overall upward trend of a net increase in THA revision procedures from 2001 to 2015 reported in our study is consistent with trends observed in other western countries [2, 5, 10, 12, 20–24]. Although AOA did observe an overall increase in THA revisions, the proportion of hip replacements that are revisions has decreased [23].This decrease in proportion of hip replacements that are revisions was also observed in Sweden [20]. The data at our disposal did not allow us to determine the percentage of total hip replacements that were revisions.

Medical advancements which have increased life expectancy and higher levels of activity in old age can be expected to have contributed to the increased demand for THA revisions [5]. Surgical indications for primary THA are extending to younger patients and considering the average life span of a prosthesis, estimated to be between 15 and 20 years, an increase in THA revision surgeries can be expected [3, 5, 6, 10, 11, 25]. Understanding the causes for revision is essential in identifying avoidable complications during primary implantation to decrease the revision burden.

Some limitations in this study should be noted. First, our study was limited in investigation of comorbidities and sex effects on the causes for THA revision. Additionally, our study did not look into how the type of revision and hospital could affect patient’s LOS. Finally, we were unable to look at the time elapsed after primary THA as a risk factor for THA revision. Further investigation into these three areas could be useful in identifying patients at increased risk of THA revision as well as help with resource allocation. We were limited in our ability to investigate these factors by the data at our disposal. Strengths of this study include its specific investigation into THA revisions in Italy, for which there is little literature available. Additionally, analysis of the causes of THA is valuable in painting a complete picture of the epidemiology of THA revisions. Finally, our consideration of LOS is not only useful for observing trends in post-operative care but also in determining resource allocation planning for patient stays.

Mechanical complications of the internal orthopedic device implant and graft, mechanical loosening of the prosthetic joint and infection and inflammatory reaction due to internal joint prosthesis were found to represented the main causes of reoperation. These findings are consistent with previous studies which found the three most common indications for THA revision were aseptic loosening, infection, and dislocation [10, 12, 14, 15, 19–21, 23, 24, 26]. The actual understanding of the causes of reoperations is complicated due to the way data is reported by surgeons in orthopedic clinics.

Prior to 2005, the general trend was to attribute to all patients with a failed arthroplasty the general code ICD-9-CM 996.4, complication of an internal orthopedic device, without further details regarding the characteristics of the failure. Likewise, all THA revision surgeries were indicated with ICD-9-CM 81.53, revision total hip arthroplasty, regardless of the specific features of the surgical procedure. The lack of a specific differentiation between the causes and the types of THA revisions make it rather challenging to exploit the main databases for epidemiological purposes. In October 2005 a new ICD-9-CM code was introduced which assigned more specific subcodes. Our data shows that the introduction of the new code has improved the net quality of the data available, however, this improvement did not affect the data regarding the type of intervention performed. Regardless of year, the ICD-9-CM 81.53 code was the most commonly used code which does not provide particular information on the portion of the prosthesis replaced.

When examining age and sex as risk factors for THA revision our findings were consistent with previously reported data. Female patients account for a larger proportion of THA revision surgeries [6, 14, 19–22, 24, 27]. Further investigation into ethnicity could be useful as a study conducted in South Korea reported a higher rate of THA in men due to the fact that osteonecrosis of the femoral head affects mostly young male adults [7]. Our data shows male patients have a lower average age at the time of THA revision. We could argue that by considering the main causes of primary intervention in male patients, such as aseptic loosening or traumas, which tend to occur in at a younger age [14, 27]. It is likely that a first surgery performed at an active age favors an early deterioration of the prosthesis. In regard to age at primary THA as a risk factor for THA revision, increased risk of revision was found for younger patients and the risk of revision generally decreased per additional decade of age [14]. These findings are in agreement with data reported by the AOA which found that rate of revision decreases with increasing age and females < 55 have almost twice the rate of revision compared to those > 75 [21, 23]. A younger age at the time of first surgery is associated with a high risk of THA revision due to aseptic loosening but was also associated with a lower risk of dislocation [14]. The average age at the time of the second surgery was rather stable, most likely due to a balance between early diagnosis, improved surgical equipment and increased life expectancy. Our data shows that the risk of THA revision tends to increase up to the age of seventy and then decreases sharply after age eighty. The data at our disposition does not allow us to assess the impact of prothesis design or surgical approach on the risk of reintervention.

The average LOS was found to have decreased over the 14-year study period. The decrease in LOS could be the result of improved peri-operative care, but might also be a result of the attempt hospitals have made to decrease financial losses [6]. The observed decrease LOS seen in our study is consistent with trends seen in the United States and Sweden [6, 20]. Our observed average LOS of 14.78 days at the end of the study period was in-between 5.43 days in the United States, 2 days in Sweden and 25.1 days in Korea [6, 7, 20]. Most of the THA revision procedures were performed in the regions of northern Italy. It remains unclear whether the observed differences reflect variations in need or clinical practice. In conclusion, this study confirms that the revision hip replacement is growing and heavily affecting the old population (mainly between 65 and 89 years). Mechanical complications of the internal orthopedic device implant and graft, mechanical loosening of the prosthetic joint and infection and inflammatory reaction due to internal joint prosthesis represented the main causes of reoperation. The average LOS was found to have decreased over the 14-year study period. Understanding the causes and risk factors for revision is essential in identifying avoidable complications and improving preventative care for patients undergoing primary implantation to decrease the revision burden. Further investigation into additional comorbidities and risk factors should be undertaken to better understand what type of patient is at risk for THA revision.

## Conclusions

Revision hip replacement is growing and heavily affecting the population between 65 and 89 years and the main causes of THA revision have been quantified. The average LOS was found to have decreased over the 14-year study period. Understanding the causes and risk factors for revision is essential in identifying avoidable complications and improving preventative care for patients undergoing primary implantation to decrease the revision burden.

## Data Availability

The datasets analysed during the current study are not publicly available due to privacy reason but are available from the corresponding author on reasonable request.

## Abbreviations

THA: Total hip arthroscopies
SDO: The National Hospital Discharge reports
RHR: Revision Hip Replacements
LOS: Length of hospitalization
ICD-9-CM: The International Classification of Diseases, Ninth Revision, Clinical Modification
ISTAT: The National Institute for Statistics
SPSS: The Statistical Package for Social Sciences
